# On Training

**Published:** 1881-12

**Authors:** 


On Training.—The one who can keep a cool head in times of excitement always has the advantage. It is sometimes mistaken for indifference, but it is a very different trait of character. Mothers, in training their children, should begin as soon as possible to teach this to a child. Temper, if allowed to go ungoverned, soon holds complete sway. Men, in training animals, take care that nothing shall make them vicious or ugly tempered; but how many think of that in training a child? How long would a horse stand quiet if some one stood behind and constantly goaded him with a small, sharp instrument? And yet children are expected to be as amiable as possible under a constant fire of nagging, teasing and a parent's ill humor. Days go by without a kind word spoken to them. When asked to do things, it is a rough command. Yet they are expected to be deferential, obedient, careful, thoughtful and kind in all their address to a parent.

A burglar entered a house the other night, and scared a lady so badly that her hair, which was lying in an exposed place on the bureau, turned white in a single night.

